# Vertical dispersal of *Aedes albopictus* within multi-story buildings in downtown Shanghai, China

**DOI:** 10.1186/s13071-023-05732-1

**Published:** 2023-06-01

**Authors:** Shuqing Jin, Jian Fan, Hui Cao, Zhendong Zhang, Peien Leng, Qiang Gao

**Affiliations:** 1 Department of Vector & Parasite Control, Huangpu Center for Disease Control & Prevention, Shanghai, 200023 People’s Republic of China; 2 Department of Vector & Parasite Control, Shanghai Municipal Center for Disease Control & Prevention, Shanghai, 200336 People’s Republic of China

**Keywords:** *Aedes albopictus*, Vertical dispersal, Mosq-ovitrap, Human landing catch, Multi-story building, Downtown Shanghai

## Abstract

**Background:**

Shanghai has numerous high-rise apartment and office buildings, but the effects of these high-rise spaces on the vertical dispersal, oviposition and blood feeding behavior of *Aedes albopictus* are unknown.

**Methods:**

In six multi-story building blocks in downtown Shanghai, 174 mosq-ovitraps (MOT) were placed both indoors and outdoors for *Ae. albopictus* collection at different vertical heights from the 1st to 6th floors and a terrace on the 8th floor. Collections were made for 4 months. The human landing catch (HLC) method for *Ae. albopictus* monitoring was also conducted on 6 consecutive days on six floors of two of the six buildings to study the feeding behavior of *Ae. albopictus* at different heights.

**Results:**

Both MOTs and HLCs collected *Ae. albopictus* at all monitored heights. The vertical distribution, oviposition pattern and biting behavior varied significantly among the seven heights (1st–6th floors and 8th floor) (mosq-ovitrap index (MOI): *X*^*2*^ = 140.616, *df* = 6, *P* < 0.001; HLC: *F *_*(5, 138)*_ = 15.111, *P* < 0.001). The MOI at low heights (1st + 2nd floors) was significantly higher than that at medium (3rd + 4th floor, *P* < 0.001) and high heights (5th + 6th floors, *P* < 0.001), and there was no significant difference in the MOI for the 3rd–6th floors. The outdoor MOIs were significantly higher than indoor MOIs at all heights (outdoor 23.09% vs. indoor 9.58%, *X*^*2*^ = 74.121, *df* = 1, *P* < 0.001). *Aedes albopictus* HLC density on the ground floor was significantly higher than that on all other heights (5.04 vs. 0.13, 0.29, 0.58, 0.79 and 1.50 per half hour, *P* < 0.05), while no difference was detected among the heights above the ground floor (*P* > 0.05).

**Conclusions:**

*Aedes albopictus* is more common near the ground level, but it can easily disperse to higher floors in the multi-story buildings of urban Shanghai. No significant differences in *Ae. albopictus* density were detected within the 3rd–6th floors using MOT or HLC. This suggests that *Ae. albopictus* might also disperse to areas above the 6th floor and seek hosts there. *Aedes albopictus* prefers to oviposit outdoors; however, *Ae. albopictus* was also able to inhabit, oviposit and engage in blood-feeding behavior indoors on different floors. The three-dimensional dispersal pattern of *Ae. albopictus* in urban areas could facilitate arbovirus transmission and increase the difficulty of dengue control.

**Graphical abstract:**

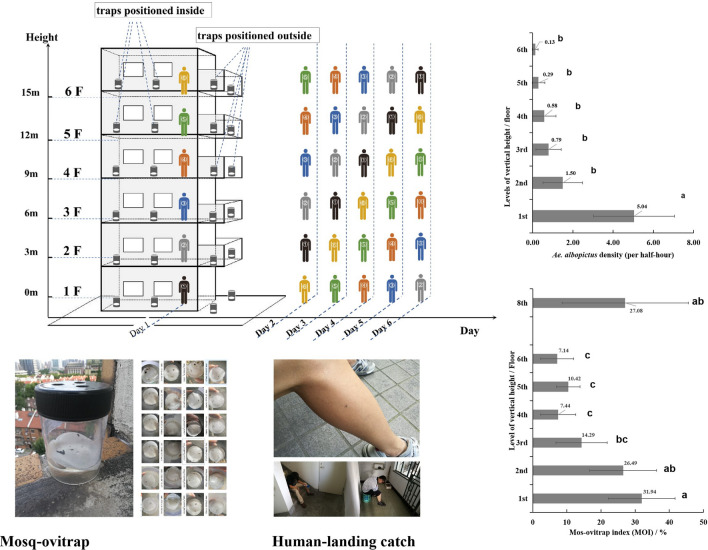

## Background

*Aedes albopictus*, the Asian tiger mosquito, is the most medically important insect vector species in Shanghai, China. It is especially important because of its role in the transmission of diseases including dengue [1, 2]. *Aedes albopictus* is an aggressive human biting species and a public health threat. It is currently a major priority for control efforts, especially since the first autochthonous dengue case was reported in Shanghai [3].

To improve mosquito surveillance, control and management practices, the understanding of mosquito biology must be improved [4]. An important aspect of mosquito biology is the vertical flight dispersal range of the mosquito. Knowledge of the vertical movements of mosquito vectors aids in the understanding of disease transmission dynamics and helps determine the controls needed to interrupt pathogen transmission [5]. This is particularly important in highly urbanized areas such as Shanghai. Many studies have evaluated the vertical oviposition and distribution patterns of mosquitoes occupying equatorial forests and savannahs [6], but relatively little attention has been paid to mosquito problems in urban areas.

*Aedes albopictus* is the dominant, and most important, vector in Shanghai. This species prefers to oviposit at the ground level, but *Ae. albopictus* can alter their movements to adapt to decreased habitat availability, leading to more opportunistic oviposition behavior [7]. *Aedes albopictus* has been found to oviposit at 3.5 m, 6 m and 7 m in Sri Lanka and Louisiana (USA) [8, 9]. Another study showed that *Ae. albopictus* could move vertically through a 60-m-high apartment building and oviposit at all heights [10]. In Shanghai, and other locations in China, knowledge of *Ae. albopictus* oviposition at different heights is lacking.

In addition to resting or seeking oviposition sites, the active vertical dispersal of *Ae. albopictus* can also be triggered by the search for mates and blood meal hosts. Many structural changes have occurred within the downtown area of Shanghai including an increase in the number of high-rise apartment and office buildings. Over 80% of the population lives in high-rise apartments in downtown Shanghai, and *Ae. albopictus* often occurs in residences located in high-rise apartments. However, no study has evaluated the impact of the construction of high-rise residences on the behavior or adaptation of *Ae. albopictus* in Shanghai. There is little information in the literature about the vertical dispersal of *Ae. albopictus* when it seeks hosts for blood meals.

The ovitrap or mosq-ovitrap (MOT) is an efficient and sensitive device for detecting the presence/absence of dengue vectors, even at low population densities [11]. The human landing catch (HLC) method is controversial in that it may pose a risk to monitoring participants. However, HLC is the most effective method for adult *Aedes* sampling. The present study used both the MOT and HLC methods to evaluate the vertical dispersal of *Ae. albopictus* host-seeking and ovipositing behavior in six high-rise apartments with ≥ 6 stories in an urban environment. This is the first study in China to describe the vertical dispersal and distribution of *Ae. albopictus* in multi-story buildings.

## Methods

### Study sites

The study was conducted within the downtown areas of Shanghai China (31°13′N, 121°27′E, elevation 3.5 m). Six building blocks ≥ 6 stories located in five residential neighborhoods and one enterprise campus were selected for mosquito sampling. Geographical and ecological descriptions of the study sites are given in Table 1.

**Table 1:** Geographical and ecological information of the six building blocks

Site ID	Type of environment	Building heights	Target floors with traps	No. of MOTs^a^ placed	Coordinates	Ecological description
Site 1	Residential neighborhood	> 20 floors	1–6F	26	31°12′30.52"N, 121°28′28.41"E	A high-rise residential building located in a well-greened residential neighborhood. There are many green spaces on the ground, and residential area is adjacent to a demolition site, where there are many potential breeding grounds for mosquitoes
Site 2	Residential neighborhood	8 floors	1–6F, 8F^b^	16	31°12′58.66"N, 121°27′21.21"E	A 90-year-old residential building located near a street intersection. There is not much green around the building, but there is a terrace on the 8th floor of the top floor, on which some potted plants are cultivated, and there are water tanks and other water storage containers
Site 3	Residential neighborhood	10 floors	1–6F	24	31°12′21.48"N, 121°28′56.92"E	A medium-height building located in a well-greened residential neighborhood with a good public environment and sanitation
Site 4	Residential neighborhood	> 20 floors	1–6F	48	31°13′27.99"N, 121°29′41.63"E	A high-rise building located in a large-scale residential neighborhood with dense buildings and a low greening rate
Site 5	Residential neighborhood	> 20 floors	1–6F	48	31°12′55.88"N, 121°28′55.00"E	A high rise building located in a large-scale residential neighbourhood with dense buildings and a low greening rate
Site 6	Enterprise and institution	12 floors	1–6F	12	31°12′12.97"N, 121°27′23.76"E	A medium-height building located in an enterprise campus. The campus has only this single building, and there is a large green area on the ground floor

^a^MOTs: mosq-ovitraps

^b^In Site 2, in addition to the 1st–6th floors, a terrace on the 8th floor was also included for MOT monitoring

### Mosquito sampling

Mosquito monitoring was conducted using two sampling methods: MOT and HLC. MOT was carried out for four months from 21 June to 20 October 2019, and HLC was conducted for 6 consecutive days in August 2019. During the 4 months, the average monthly maximum temperature ranged from 23 ℃ to 32 ℃, and there were 26 days of precipitation, with the most precipitation occurring in August. There was no rain in the 6 days when the HLC was performed.

### Mosq-ovitrap (MOT)

The MOT (Tianpai, Kaiqi Co. Ltd, Shanghai, China) as described by Lin [12] and Gao [11] was used in this study. The MOT is a modified ovitrap with a design for easy mosquito entry but a difficult exit. The MOT used in this study consists of a transparent cylindrical plastic jar with a black top cover with three conical holes. A circular white filter paper (7.5 cm in diameter) is placed at the bottom of the jar as an oviposition substrate, and 20 ml dechlorinated tap water is added to the jar to keep the filter moist but not submerged. The MOT can collect both adult mosquitoes seeking oviposition sites and eggs produced by trapped females.

In this study, MOT sampling was conducted for 4 months, with 10 days as a sampling interval. A fixed number of MOTs were placed on each floor covering the 1st–6th floors of the six buildings and a terrace on the 8th floor in Site 2 (total 174 traps × 12 sampling intervals = 2088 trap times). The number of MOTs placed at different monitoring building was not the same, which mainly depended on the public area size of the floor. On average, one MOT was placed every 10 m². The locations of MOTs on each floor were divided into indoor and outdoor environments according to the straight line distance from a window or outside. On each floor, “outdoor” in this study refers to balconies projecting outward, open windowsills or areas with a distance < 0.5 m from windows. “Indoor” refers to other interior spaces of the apartment block such as corridors, stairways and under shoe racks located next to doors. To maximize accessibility and operability, MOTs were not placed inside the homes of the occupants.

For each 10-day sampling interval, the number of adult mosquitoes trapped and the number of eggs produced and trap conditions (i.e., tipped, dried, missing, flooded or broken) were recorded. Collections were made between 09:00 and 12:00 h, which is the daily time with the lowest mosquito activity [13]. A positive MOT was defined as a trap containing at least one adult, egg or larva.

Trapped mosquitoes were collected and stored at − 80 ℃.

### Human landing catch sampling

**Fig. 1:**
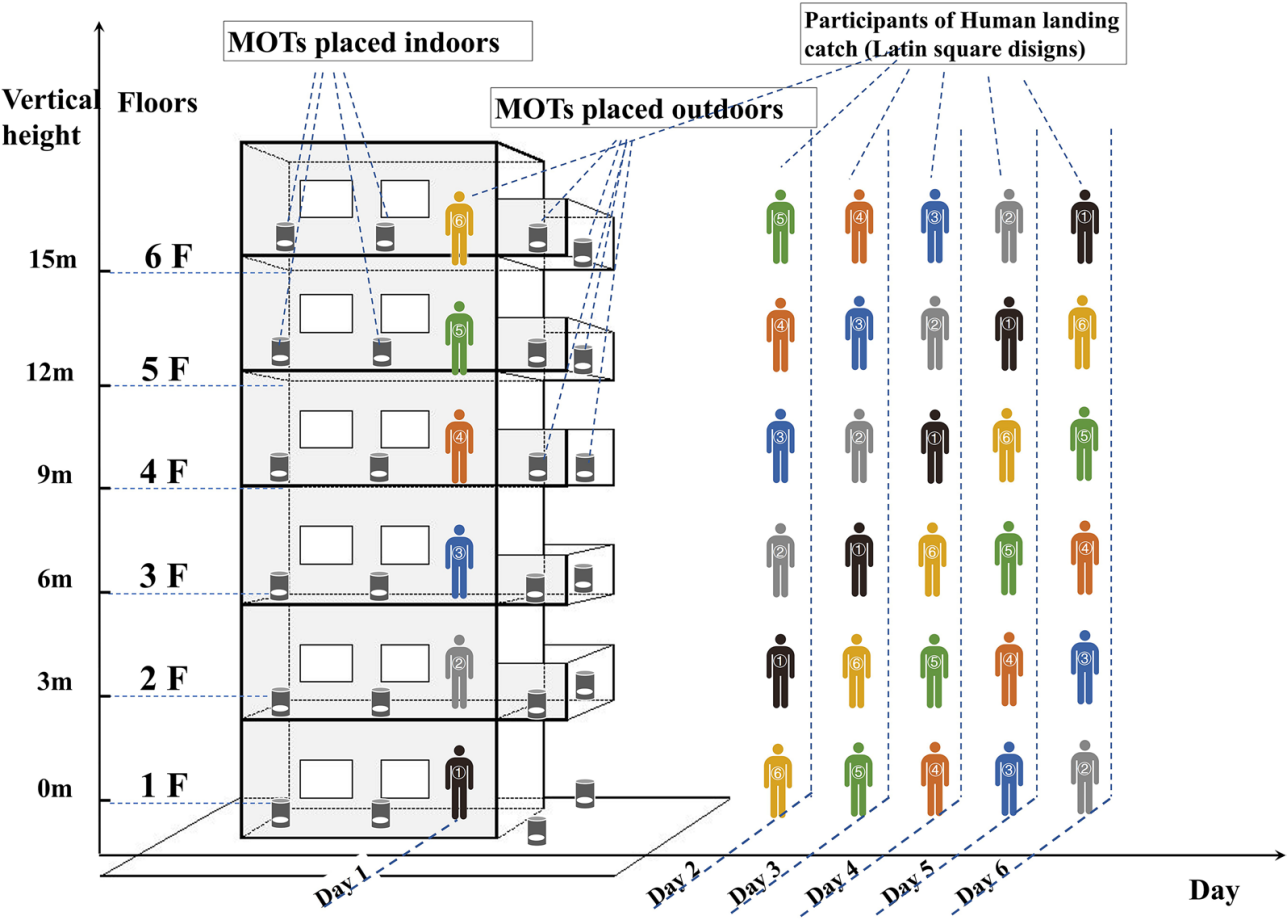
Vertical monitoring of *Aedes albopictus* on six floors of different heights using the mosq-ovitrap (MOT) and human landing catch (HLC) sampling methods

HLC sampling was conducted for 6 consecutive days in early August of 2019, at heights from the 1st floor to the 6th floor in two buildings located in Site 4 and Site 5. A total of 12 volunteers aged between 31 and 59 years were recruited and trained for HLC after their informed content was obtained. The participants were divided into two groups, with six volunteers designated for each building. Within each group, one participant occupied each floor from the 1st–6th floor. The participants stood still and collected mosquitoes landing on their exposed arms or legs using a portable, battery-powered aspirator. The catch period had a duration of 30 min and was performed twice a day, including in the early morning (7:30–8:00 h) and during the late afternoon before sunset (16:30–17:00 h). To eliminate human-bait attraction bias caused by individual differences among the volunteers, a Latin-square design was adopted for HLC sampling in each building. For each of the 6 consecutive days, each individual volunteer occupied a different floor on a different day (Fig. 1).

The HLCs were conducted in areas without reported dengue or Zika cases. According to the local center for disease control and prevention (CDC), there were no other *Aedes*-vectored disease cases during the study period.

### Mosquito processing

Adult mosquitoes trapped by MOTs and HLCs were killed by freezing and then counted and identified using taxonomic keys [14]. Eggs or eclosed larvae collected by MOTs after 10 days were returned to the laboratory. For species identification, we randomly selected 20% of the trapped eggs or larvae and then hatched and reared these until adult emergence. Adults were identified, and the results were extrapolated to the remaining unhatched eggs.

In the laboratory, the eggs were hatched by immersing them in transparent glass pans filled with Milli-Q water. Larvae were reared using standard techniques, and adults were identified based on taxonomic keys [14] in the same manner used for MOTs and HLCs.

### Statistical methods

All data obtained from this study were analyzed as follows:

i. Mosq-ovitrap index (MOI): the percentage of positive MOTs against the total number of MOTs recovered from each monitoring site;
ii. Adult *Ae. albopictus* density: the mean number of *Ae. albopictus* per recovered positive MOT or sampled using HLC per 30 min;
iii. Egg density: the mean number of eggs per recovered positive MOT.
iv. Among the 2088 MOTs, the number of invalid MOTs caused by loss or other reasons was small (*n* = 11). For these missing values, the average values of the remaining MOTs on the same floor in the same period were used as a replacement.

Data were analyzed using the SPSS version 13.0 (SPSS, Inc., Chicago, IL, USA) statistical package. For the MOI percentage data, Chi-square was used for comparison. Quantitative data, such as mosquito and egg density, were not normally distributed, After logarithmic transformation of the data, an independent t-test or one-way analysis of variation (one-way ANOVA) was used for comparison, and Tukey’s test was used for pairwise comparison. Pearson correlation analysis was used for the vertical distribution of the mosquito sampling yields of MOT and HLC. A value of *P* < 0.05 was considered to represent a statistically significant difference.

## Results

### Mosquito population structure

A total of 9245 eggs were collected using the MOTs. A randomized 20% sample of these eggs was reared to adults, and all were identified as *Ae. albopictus*. A total of 481 adult *Ae. albopictus* (♀: ♂ = 355: 15), two adult female *Culex pipiens* complex and one adult female *Cx. tritaeniorhynchus* were collected using the MOTs. Two species were collected by HLCs, including 200 *Ae. albopictus* (♀: ♂ = 180: 20) and two female *Cx. pipiens* complex. The female *Culex* mosquitoes were mostly collected at the ground level (only one was collected on the 6th floor) and were scattered in different sites. It is not within the scope of this paper to present the results for other mosquito species; therefore, the results are limited to *Ae. albopictus*.

### Vertical and temporal distribution of *Ae. albopictus* with MOTs

Adult female *Ae. albopictus*, or their eggs, were found in MOTs placed at all heights from the ground floor to the 6th floor in all six buildings. The overall number of positive MOTs was 349 (MOI = 16.71%). Among the positive MOTs, the mean densities of adult *Ae. albopictus* and eggs were 1.39 and 26.49 per trap, respectively.

The *Ae. albopictus* density and oviposition patterns monitored using the MOTs in the six study sites are shown in Table 2. No significant differences were detected in the two parameters of adult *Ae. albopictus* density and egg density among the six sites (*P* = 0.107; *F*_*(5, 343)*_ = 1.140, *P* = 0.349; *F*_*(5, 343)*_ = 0.679, *P* = 0.641), However, the MOI varied significantly among the six sites (*X*^*2*^ = 23.498, *df* = 5, *P* < 0.001), which was attributed to the environmental differences among sites.

**Table 2:** Spatial comparison of *Aedes albopictus* collected using MOTs at six study sites

Site	MOT traps·time^a^	Positive traps		Adult *Ae. albopictus*		Eggs of *Ae. albopictus*	
		No. of positive traps·time	Mosq-ovitrap index/% (95% CI)	No. of *Ae. Albopictus* collected	Density (per positive trap) (95%CI)	No. of eggs collected	Density (per positive trap) (95% CI)
1	312	45	14.42 (10.5, 18.34)	67	1.51 (1.07, 1.94)	1274	28.31 (12.47, 44.15)
2	192	19	9.9 (5.63, 14.16)	21	1.11 (0.88, 1.33)	400	21.05 (9.89, 32.21)
3	288	51	17.71 (13.27, 22.14)	56	1.11(0.76, 1.47)	1150	22.55 (16.15, 28.94)
4	576	84	14.58 (11.69, 17.47)	120	1.43 (1.14, 1.71)	2494	29.69 (21.49, 37.89)
5	576	129	22.4 (18.98, 25.81)	192	1.50 (1.32, 1.67)	3369	26.12 (20.71, 31.52)
6	144	21	14.58 (8.75, 20.42)	25	1.19 (0.72, 1.65)	558	26.57 (15.12, 38.02)
Sum	2088	349	16.71 (15.11, 18.32)	481	1.39 (1.26, 1.51)	9245	26.49 (22.86, 30.12)
Chi-square test /One way ANOVA			X^2^ = 23.498 *P* < 0.001		F = 1.214 *P* = 0.302		F = 0.396 *P* = 0.851

^a^MOTs: mosq-ovitraps; the number of mosq-ovitraps was different among different sites, which depended on the the public area size of the floor

**Fig. 2:**
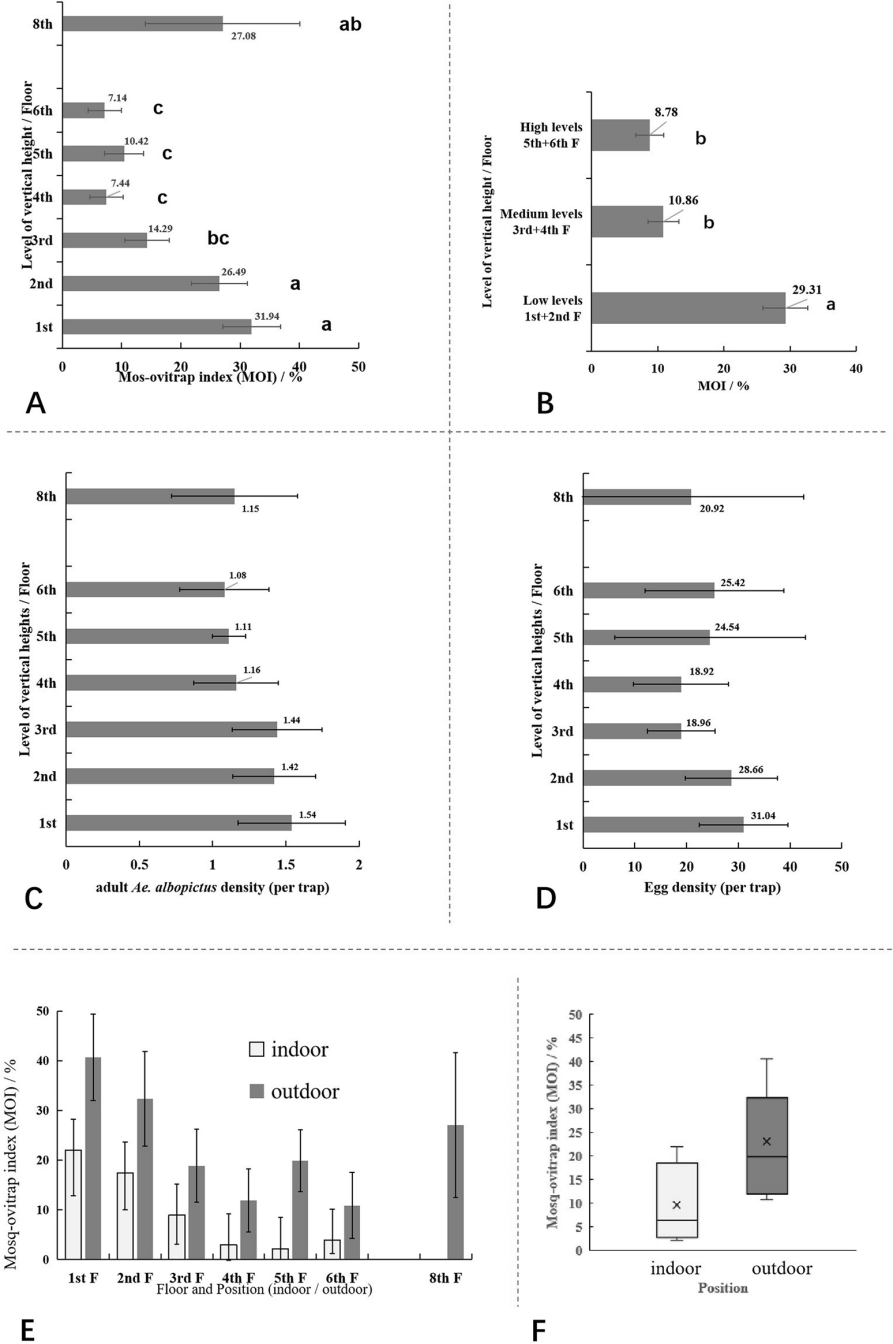
Vertical distribution and indoor/outdoor preference of *Aedes albopictus* sampled using the mosq-ovitrap (MOT) in downtown Shanghai. **A**, **B** Comparison of the mosq-ovitrap index (MOI) at different vertical heights (lower case letters a, b and c indicate statistically significant difference at *P* < 0.05, using the Chi-square test for comparison, and the dispersion bars mean 95% CI). **C**, **D** Comparison of adult *Ae. albopictus* density and egg density in positive MOTs at different vertical heights (the dispersion bars mean 95% CI). **E**, **F** Comparison of MOI values indoors and outdoors (the dispersion bars mean 95% CI)

The *Ae. albopictus* vertical distribution and oviposition patterns varied significantly among the seven floors (1st–6th floors and 8th floor) (MOI: *X*^*2*^ = 140.616, *df* = 6, *P* < 0.001) (Table 3). Pairwise comparison showed that the MOI values at the ground level (1st floor) and the 2nd floor were significantly higher than the MOIs of the 3rd, 4th, 5th and 6th floors (Fig. 2A). After regrouping, the MOI values at low heights (1st + 2nd floors) were significantly higher than those at medium heights (3rd + 4th floor, *P* < 0.001) and high heights (5th + 6th floors, *P* < 0.001). There was no significant difference in the MOI values between the medium and high floors (*P* = 0.802) (Fig. 2B).

**Table 3:** Vertical distribution of *Aedes albopictus* sampled using MOTs at different floor heights

Vertical heights/floors (m)	MOT traps·time	Positive traps		Adult *Ae. albopictus*		Egg of *Ae. albopictus*	
		No. of positive traps·time	Mosq-ovitrap index/% (95% CI)	No. of *Ae. Albopictus* collected	Density (per positive trap) (95% CI)	No. of eggs collected	Density (per positive trap) (95% CI)
1st F (0 ~ m)	360	115	31.94 (27.1, 36.78)	177	1.56 (1.29, 1.82)	3570	31.04 (23.12, 38.96)
2nd F (3 ~ m)	336	89	26.49 (21.75, 31.23)	126	1.42 (1.15, 1.68)	2551	28.66 (21.02, 36.30)
3rd F (6 ~ m)	336	48	14.29 (10.52, 18.05)	69	1.44 (1.08, 1.79)	910	18.96 (11.91, 26.00)
4th F(9 ~ m)	336	25	7.44 (4.62, 10.26)	29	1.16 (0.93, 1.39)	473	18.92 (9.52, 28.32)
5thF (12 ~ m)	336	35	10.42 (7.13, 13.7)	39	1.11 (0.95, 1.28)	859	24.54 (15.67, 33.41)
6thF (15 ~ m)	336	24	7.14 (4.38, 9.91)	26	1.13 (0.82, 1.44)	610	25.42 (15.58, 35.25)
8thF (21 ~ m)	48	13	27.08 (14.04, 40.12)	15	1.15 (0.81, 1.49)	272	20.92 (6.86, 34.99)
Sum	2088	349	16.71 (15.11, 18.32)	481	1.39 (1.26, 1.51)	9245	26.49 (22.86, 30.12)
Chi-square test/One way ANOVA			X^2^ = 140.616 *P* < 0.001		F = 1.174 *P* = 0.319		F = 1.053 *P* = 0.391

**Table 4:** Spatial and temporal distribution of *Aedes albopictus* monitored using the HLC method at different vertical heights at Site 4 and Site 5

Level of vertical height/floor	Early morning (7:30–8:00 h)		Late afternoon before sunset (16:30–17:00 h)	
	No. of adult *Ae. albopictus* collected	Density/per 30 min (95% CI)	No. of adult *Ae. albopictus* collected	Density/per 30 min (95% CI)
Site 4				
1	19	**3.17** (− 0.55, 6.89)	35	**5.83** (0.72, 10.95)
2	2	0.33 (− 0.21, 0.88)	27	**4.50** (1.33, 7.67)
3	3	0.50 (− 0.38, 1.38)	15	**2.50** (0.22, 4.78)
4	5	0.83 (− 0.85, 2.51)	9	**1.50** (− 0.57, 3.57)
5	1	0.17 (− 0.26, 0.60)	6	**1.00** (− 0.33, 2.33)
6	0	0 (0, 0)	2	0.33 (− 0.21, 0.88)
Sum	30	0.83 (0.20, 1.47)	94	**2.61** (1.53, 3.69)
Site 5				
1	16	**2.67** (− 0.42, 5.76)	51	**8.50** (2.50, 14.50)
2	3	0.50 (− 0.07, 1.07)	4	0.67 (− 0.19, 1.52)
3	0	0 (0, 0)	1	0.17 (− 0.26, 0.60)
4	0	0 (0, 0)	0	0 (0, 0)
5	0	0 (0, 0)	0	0 (0, 0)
6	0	0 (0, 0)	1	0.17 (-0.26, 0.60)
Sum	19	0.53 (0.02, 1.04)	57	**1.58** (0.29, 2.88)

For positive MOTs, the density of adult *Ae. albopictus* and eggs was relatively balanced and no significant difference was detected among among the seven floors (1st–6th floor and 8th floor) (adult *Ae. albopictus*: *F*_*(6, 342)*_ = 1.030, *P* = 0.415; egg density: *F*_*(6, 342)*_ = 1.278, *P* = 0.281).

After removing the data for the 8th floor of Site 2, there was a significant negative correlation between the MOI values and the vertical height levels (Pearson r _*(35)*_ =  − 0.605, *P* < 0.001). For the number of *Ae. albopictus* adults and eggs collected, there was a logarithmic trend in relation to the floors (*Ae. albopictus*: y =  − 90.32ln(x) + 176.7, R^2^ = 0.952; *Ae. albopitctus* eggs: y =  − 1795ln(x) + 3463.4, R^2^ = 0.8844; “x” means floor number). This trend suggests that the *Ae. albopictus* density decreases with the increase of vertical heights, but the extent of the decrease also decreases with the rise of the vertical heights.

For the indoor/outdoor effect on *Ae. albopictus* dispersal and oviposition behavior at vertical heights, it was observed that *Ae. albopictus* preferred to oviposit outdoors. The MOI values of outdoors were significantly higher than the indoor values at all heights (1st floor to 6th floor) (outdoor 23.09% vs. indoor 9.58%, *X*^*2*^ = 74.121, *df* = 1, *P* < 0.001) (Fig. 2E and F). The effect of vertical floor heights and indoor/outdoor variance was studied using a two-way ANOVA; there were no significant differences in adult *Ae. albopictus* and egg densities between indoors and outdoors (*Ae. albopictus*: outdoor 1.28 vs. indoor 1.25 per trap, *F *_*(1,347)*_ = 0.906, *P* = 0.342; eggs: outdoor 23.78 vs. indoor 29.32 per trap, *F *_*(1,347)*_ = 0.455, *P* = 0.501), and no significant difference was found among different floors.

**Fig. 3:**
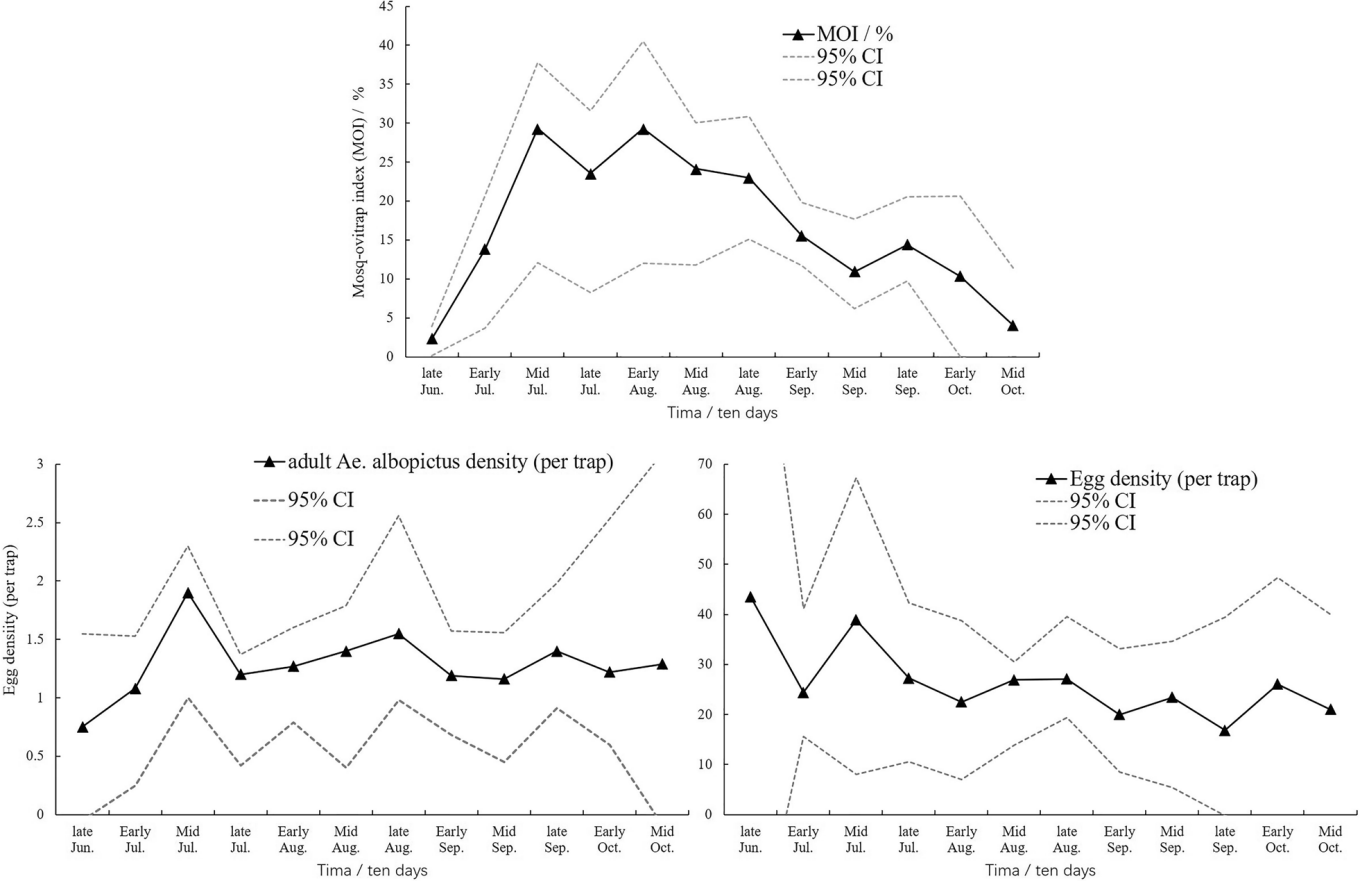
Temporal distribution of *Aedes albopictus* in the six experimental buildings in downtown Shanghai

The temporal distributions of *Ae. albopictus* at all seven heights monitored using MOTs are shown in Fig. 3. The temporal variances of adult *Ae. albopictus* and egg density were not significant (*F*_*(11, 337)*_ = 1.657, *P* = 0.109; *F*_*(11, 337)*_ = 0.822, *P* = 0.619). The MOI values fluctuated greatly in different months. The MOI values peaked in mid-July and early August (29.31% and 29.31%, respectively), while in late June and mid-October, the MOIs were < 10%.

### Human landing catch

In Sites 4 and Site 5, a total of 144 HLCs time (six participants × two buildings × two 30-min periods per day × 6 days) were performed over 6 consecutive days. The mean density of adult *Ae. albopictus* in the late afternoon was significantly higher than the adult density during the early morning (2.10 vs. 0.68 per 30 min, *t *_*(143)*_ =  − 3.065, *P* = 0.003) (Table 4).

**Fig. 4:**
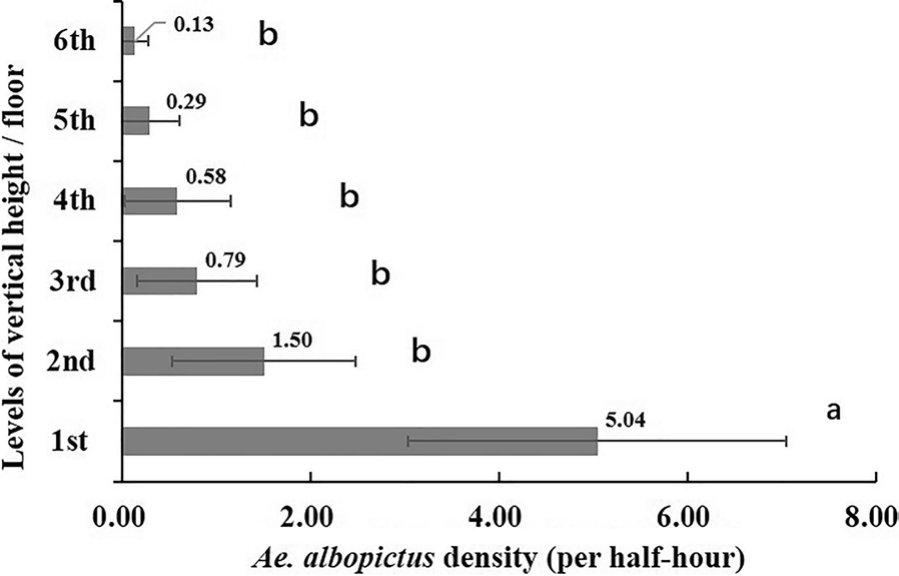
Vertical distribution of *Aedes albopictus* collected using the human landing catch (HLC) method in two sites in downtown Shanghai (lowercase letters a and b indicate statistically significant difference at *P* < 0.05, using Tukey’s test for pairwise comparison between different heights; the dispersion bars mean 95% CI)

Adult *Ae. albopictus* were collected at every vertical height from the ground floor to the 6th floor at Site 4, while *Ae. albopictus* were only collected on the 1st, 2nd, 3rd and 6th floors at Site 5. The vertical distribution of *Ae. albopictus* varied significantly among the different levels (*F *_*(5, 138)*_ = 15.111, *P* < 0.001), and the highest density of *Ae. albopictus* was found on the ground floor at Site 4 and Site 5. There was also a a logarithmic trend between the *Ae. albopictus* density and the height of the floors (y = − 2.608ln(x) + 4.2489, R^2^ = 0.8703), indicating that *Ae. albopictus* density decreased with the increase of vertical height, but the extent of the decrease also decreased with the rise of the vertical heights. Pairwise comparison using the Tukey method showed that the density of *Ae. albopictus* on the 1st floor was significantly greater on the 1st floor than on the 2nd−6th floors (5.04 vs. 0.13, 0.29, 0.58, 0.79 and 1.50 per 30 min, *P* < 0.05). No significant difference in *Ae. albopictus* density was detected among the heights above the ground floor (*P* > 0.05) (Fig. 4).

## Discussion

People living or working in the high-rise buildings of Shanghai often complain about being bitten by mosquitoes. There is a need for up-to-date information on *Ae. albopictus* vertical dispersal in urban areas to guide control methods [10].

The field monitoring used in this study confirmed that *Ae. albopictus* were present, ovipositing and seeking blood meal hosts on the 1st–6th floor heights in all of the monitored buildings in downtown Shanghai. Many *Ae. albopictus* were also found breeding and flying on an 8th floor terrace. *Aedes albopictus* were typically more common in areas near the ground, such as the ground floor and the 2nd floor in this study. However, it appears that they can readily disperse to the higher floors of multi-story buildings. In areas between the height range of the 3rd–6th floors, there was no significant difference in *Ae. albopictus* density, as monitored using the MOI. As an exophilic species, *Ae. albopictus* preferred to oviposit in the outdoor areas on different floors. The MOI in outdoor environments was significantly higher than that in indoor environments. However, *Ae. albopictus* can also occur and oviposit indoors. The biting rate of *Ae. albopictus* in Shanghai was higher in the evening before sunset than in the morning.

Each mosquito population has a different range of dispersal as a consequence of its intrinsic flight capacity and the ecological setting [5], and *Ae. albopictus* appears to conform to this rule. For vertical dispersal, previous studies have shown that *Ae. albopictus* prefer to fly at ground level or at heights < 3 m [15, 16]. This may be because: (i) *Ae. albopictus* prefer to oviposit in small pockets of container water, and these breeding habitats are more common on the ground; (ii) *Ae. albopictus* prefer to bite humans. In contrast to ornithophilic mosquito species that usually fly higher, anthropophilic mosquito species usually fly lower because their sugar sources and blood meal hosts are mostly distributed and active on the ground; (iii) *Ae. albopictus* appear to have relatively weak flight capacity and their maximum horizontal flight range is only 100–200 m [5, 17, 18].

In the present study, it was found that *Ae. albopictus* in urban Shanghai preferred to be active at ground level or on low building floors. It was also confirmed that these mosquitoes could disperse to higher areas, such as floors 3–6 or floor 8, and engage in blood feeding and oviposition on the higher floors. A report of *Ae. albopictus* invading higher spaces was previously documented. Amerisinghe and Alagoda [8] demonstrated that *Ae. albopictus* could oviposit at heights of 3.5 and 6 m in Sri Lanka [8]. In Louisiana, *Ae. albopictus* were found to oviposit at heights up to 7 m [9]. In Singapore, Liew and Curtis found that *Ae. albopictus* could move vertically through a 60-m-high apartment building and oviposit at all heights [10]. In Malaysia, *Ae. albopictus* were found up to the 6th floor (16.1–18.0 m), which indicated that *Aedes* could be found at even higher levels of high-rise apartments [19]. Lau et al. reported *Ae. albopictus* in Malaysia breeding up to the 14th floor (39.1–42.0 m). This indicated that *Aedes* could breed at every level of the apartment building and were not restricted by the height of the apartment [20]. This dispersal to higher areas is not limited to *Ae. albopictus* but has also been found in populations of *Aedes aegypti*, which have been found breeding on the highest floors (16th floor, 45.1–48.0 m) in some studies [20].

Marini [5] suggests that the active dispersal of *Ae. albopictus* is triggered by the search for mates, sugar sources and resting sites. For females, dispersal helps identify hosts for blood meals and oviposition sites and depends on environmental thresholds, such as light intensity, wind speed and direction, and temperature [5]. Vavassori conducted a mark-release-recapture study and found that the active dispersal of host-seeking female *Ae. albopictus* may be greater than previously reported [21]. Chadee also reported that the adaptation of *Aedes* mosquitoes to house design evolved from ground floor areas to higher elevation apartment buildings [22].

Shanghai is the largest metropolis in eastern China, and it has undergone many environmental changes, particularly in urban sanitation, housing types and population density. The following factors have favored the expanded three-dimensional spatial distribution of *Ae. albopictus*: (i) urban sanitation campaigns have removed many scattered small waste containers and reduced the potential outdoor water breeding sites, which has forced *Ae. albopictus* to fly further or higher to locate suitable water oviposition sites; (ii) the heights of buildings in the urban areas are increasing and a greater proportion of the human population lives and works in high-rise spaces. To obtain blood meals, *Ae. albopictus* also need to disperse and adapt to high-rise areas; (iii) the application of pesticides on the ground environment forces *Ae. albopictus* to fly to higher areas to find a more suitable habitat. Tinker (1974) suggests that the movement of *Ae. aegypti* above ground level may be due to insecticide treatment at the ground level [23]; (iv) other human activities may also affect the vertical dispersal of mosquitoes. The frequent use of elevators may have carried *Ae. albopictus* into high-rise areas. The growing popularity of high-rise terrace gardens in urban areas also provides good breeding habitats for mosquitoes like *Ae. albopictus*.

*Aedes albopictus* are known as an exophilic species [24]. However, they are now commonly found in indoor spaces, especially in urban areas like Shanghai. In this study, although *Ae. albopictus* preferred to oviposit outdoors on different floors, it was also confirmed that they could inhabit, bite and oviposit indoors. This may be the first vertical dispersal test of the *Ae. albopictus* population being directly evaluated using HLC. The landing *Ae. albopictus* which were detected indoors in high-elevation areas using HLC may have originated from multiple sources including the following. First, an aboriginal *Aedes* population inhabiting the particular floor was attracted to the host at a certain monitoring time. In this study, breeding sites such as water containers or small temporary pondings caused by rain were not common in the public area on the 1st to 6th floors. Whether water containers inside residential homes are the breeding source of *Ae. albopictus* on different floors needs further confirmation. Second, ground-inhabiting *Aedes* mosquitoes, which were attracted by CO_2_ and human odors, then flew or used elevators to reach higher spaces with human hosts. Chadee (2004) also investigated indoor oviposition and biting behaviors in the West Indies and found that they increased over an extended period, which was attributed to artificial lighting and peaks in human activity within homes [22]. For the biting rhythm, the biting rate in the evening before sunset was higher than that in the morning, and the biting rate was not invested over an extended period in this study.

The active dispersal of *Ae. albopictus* on higher floors and indoor spaces may pose challenges to mosquito control and dengue fever control. Current *Aedes* mosquito and dengue control measures mainly focus on the control of adult mosquitoes near the ground and the removal of outdoor breeding sites. However, as the urban ecological settings change, certain control measures near the ground may drive more *Ae. albopictus* to seek suitable habitats at higher elevations. When there is a viremic person infected with dengue living in a high-rise residential building, the female mosquitoes inhabiting the same floor or adjacent floors would be the most problematic. These females would comprise the group most likely to bite the infected person and then spread the pathogen. The implementation of mosquito control measures at the ground level would probably not have a substantial impact on the infected mosquitoes inhabiting high-rise buildings. *Aedes albopictus* living in high-elevation spaces may become a blind spot in the control of mosquitoes and dengue fever. It is therefore necessary to consider three-dimensional mosquito control measures such as residual insecticide spraying in the building where the infected person lives.

This study evaluated the oviposition and host-seeking activity of *Ae. albopictus* in urban high-rise apartments. This is the first attempt to evaluate the vertical dispersal of *Ae. albopictus* in multiple-story buildings in China, and it provides information that could facilitate the dengue vector control program in this area.

There were some limitations in the research design:

(i) As an exploratory study, investigations were limited to the 1st–6th floors. The vertical dispersal of *Ae. albopictus* in areas higher than the 6th floor should be verified in a future study. There was no significant difference in *Ae. albopictus* density monitored based on the MOI in spaces above the 2nd floor (the 3rd–6th floors), indicating that *Aedes* density may not decrease as the floor level increases. This suggests that *Ae. albopictus* can breed at every level of the apartment and may not be restricted by its height, in accordance with the suggestion of Lau [20]. There were no significant differences in egg density monitored using the MOI among different floor heights, suggesting that the vertical floor heights had no significant impact on the oviposition behavior of *Ae. albopictus*. Once *Ae. albopictus* reach a certain floor, they treat this floor as the ground starting point and then continue to spread to lower or higher floors. The activities of *Ae. albopictus* on the 7th–20th floors would be monitored as the next step in future research.
(ii) Another shortcoming is that the dispersal of *Ae. albopictus* to higher floors in this study does not verify that *Ae. albopictus* reach certain floors directly by flying. The vertical flight capacity and behavior of *Ae. albopictus* in urban areas should be further studied.

## Conclusions

Although *Ae. albopictus* are more common near the ground, they appear to easily disperse to higher floors of urban buildings. This study confirmed the presence of *Ae. albopictus* ovipositing and seeking hosts on the 1st–6th floors of multi-story buildings in urban Shanghai. No significant differences in *Ae. albopictus* density were found on the 3rd–6th floors using MOT and HLC, suggesting that *Ae. albopictus* might also disperse to spaces higher than the 6th floor and seek hosts within these spaces. *Aedes albopictus* is an exophilic species that prefers to oviposit outdoors on different floors. They can also inhabit, oviposit and seek blood meals indoors. The extensively three-dimensional dispersal pattern of *Ae. albopictus* in urban areas could facilitate arbovirus transmission and increase the difficulty of dengue control.

## Data Availability

All relevant data are within the paper.

## Abbreviations

MOT: Mosq-ovitrap
MOI: Mosq-ovitrap index
HLC: Human landing catch
ANOVA: Analysis of variance
CO_2_: Carbon dioxide

## References

[1] Gao Q, Wang F, Lv X, Cao H, Zhou J, Su F (2018). Comparison of the human-baited double net trap with the human landing catch for *Aedes*
*albopictus* monitoring in Shanghai China. Parasit Vectors.

[2] Zhou ZB, Lv S, Zhang Y, Gu WB, Guo YH, Jiang M (2015). Mosquito species, distribution and their pathogens in Shanghai, China. Chin J Vector Biol Control.

[3] Xu J, Yang Y, Sun C, Wang H, Leng P, Zhu J (2019). Evaluation on control measures and effects to *Aedes*
*albopictus* at the epidemic sites of three local occurred dengue fever inflection cases in Baoshan District of Shanghai in 2018. Chin J Hyg Insect Equip.

[4] Lee HI, Seo BY, Burkett DA, Lee WJ, Shin YH (2006). Study of flying height of culicid species in the northern part of the Republic of Korea. J Am Mosq Control Assoc.

[5] Marini F, Caputo B, Pombi M, Tarsitani G, DellaTorre A (2010). Study of *Aedes*
*albopictus* dispersal in Rome, Italy, using sticky traps in mark-release-recapture experiments. Med Vet Entomol.

[6] Snow WF (1975). The vertical distribution of flying mosquitoes (diptera, culicidae) in west african savanna. B Entomol Res.

[7] Williges E, Faraji A, Gaugler R (2014). Vertical Oviposition Preferences of the Asian Tiger Mosquito, *Aedes albopictus*, In Temperate North America. J Am Mosq Control Assoc.

[8] Amerasinghe FP, Alagoda TSB (1984). Mosquito oviposition in bamboo traps, with special reference to *Aedes albopictus*, *Aedes novalbopictus* and *Armigeres subalbatus*. Insect Sci Appl.

[9] Schreiber ET, Meek CL, Yates MM (1988). Vertical distribution and species coexistence of tree hole mosquitoes in Louisiana. J Am Mosq Control Assoc.

[10] Liew C, Curtis CF (2004). Horizontal and vertical dispersal of dengue vector mosquitoes, *Aedes aegypti* and *Aedes albopictus*, in Singapore. Med Vet Entomol.

[11] Gao Q, Cao H, Fan J, Zhang Z, Jin S, Su F (2019). Field evaluation of Mosq-ovitrap, Ovitrap and a CO2-light trap for *Aedes albopictus* sampling in Shanghai. China PeerJ.

[12] Lin LF, Lu WC, Cai SW, Duan JH, Yi JR, Deng F (2005). The design and efficacy observation of new mosq-ovitrap for monitoring of vector of dengue fever. Chin J Vector Bio Control.

[13] Reiter P, Amador MA, Colon N (1991). Enhancement of the CDC ovitrap with hay infusions for daily monitoring of *Aedes aegypti* populations. J Am Mosq Control Assoc.

[14] Becker N, Petric’ D, Zgomba M, Boase C, Dahl C, Lane J (2003). Mosquitoes and their control.

[15] Alencar J, Morone F, De Mello CF, Degallier N, Lucio PS, de Serra-Freire NM (2013). Flight height preference for oviposition of mosquito (Diptera: Culicidae) vectors of sylvatic yellow fever virus near the hydroelectric reservoir of Simplicio, Minas Gerais Brazil. J Med Entomol.

[16] Alencar J, de Mello CF, Gil-Santana HR, Guimaraes AE, de Almeida SA, Gleiser RM (2016). Vertical oviposition activity of mosquitoes in the Atlantic Forest of Brazil with emphasis on the sylvan vector, *Haemagogus leucocelaenus* (Diptera: Culicidae). J Vector Ecol.

[17] Lacroix R, Delatte H, Hue T, Reiter P (2009). Dispersal and survival of male and female *Aedes albopictus* (Diptera: Culicidae) on Reunion Island. J Med Entomol.

[18] Verdonschot P, Besse-Lototskaya AA (2014). Flight distance of mosquitoes (Culicidae): a metadata analysis to support the management of barrier zones around rewetted and newly constructed wetlands. Limnologica.

[19] Wan-Norafikah O, Nazni WA, Noramiza S, Shafa'ar-Ko'ohar S, Azirol-Hisham A, Nor-Hafizah R (2010). Vertical dispersal of *Aedes* (*Stegomyia*) spp. in high-rise apartments in Putrajaya Malaysia. Trop Biomed.

[20] Lau KW, Chen CD, Lee HL, Izzul AA, Asri-Isa M, Zulfadli M (2013). Vertical distribution of *Aedes* mosquitoes in multiple storey buildings in Selangor and Kuala Lumpur Malaysia. Trop Biomed.

[21] Vavassori L, Saddler A, Muller P (2019). Active dispersal of *Aedes albopictus*: a mark-release-recapture study using self-marking units. Parasit Vectors.

[22] Chadee DD (2004). Observations on the seasonal prevalence and vertical distribution patterns of oviposition by *Aedes*
*aegypti* (L.) (Diptera: Culicidae) in urban high-rise apartments in Trinidad West Indies. J Vector Ecol.

[23] Tinker ME (1974). *Aedes aegypti* larval habitats in Surinam. Bull Pan Am Health Organ.

[24] Delatte H, Desvars A, Bouetard A, Bord S, Gimonneau G, Vourc'h G (2010). Blood-feeding behavior of *Aedes albopictus*, a vector of Chikungunya on La Reunion. Vector Borne Zoonotic Dis.

